# Synthesis, Characterization and Kinetic Behavior of Supported Cobalt Catalysts for Oxidative after-Treatment of Methane Lean Mixtures

**DOI:** 10.3390/ma12193174

**Published:** 2019-09-27

**Authors:** Andoni Choya, Beatriz de Rivas, Jose Ignacio Gutiérrez-Ortiz, Juan Ramón González-Velasco, Rubén López-Fonseca

**Affiliations:** Chemical Technologies for Environmental Sustainability Group, Chemical Engineering Department, Faculty of Science and Technology, University of The Basque Country UPV/EHU, E-48940 Leioa, Bizkaia, Spain; andoni.choya@ehu.eus (A.C.); beatriz.derivas@ehu.eus (B.d.R.); juanra.gonzalezvelasco@ehu.eus (J.R.G.-V.)

**Keywords:** supported cobalt catalysts, spinel cobalt oxide, methane oxidation, magnesia, ceria, γ-alumina, cobalt aluminate, Co-Mg mixed oxides, oxygen mobility: Mars–van Krevelen mechanism

## Abstract

The present work addresses the influence of the support on the catalytic behavior of Co_3_O_4_-based catalysts in the combustion of lean methane present in the exhaust gases from natural gas vehicular engines. Three different supports were selected, namely γ-alumina, magnesia and ceria and the corresponding catalysts were loaded with a nominal cobalt content of 30 wt. %. The samples were characterized by N_2_ physisorption, wavelength dispersive X-ray fluorescence (WDXRF), X-ray diffraction (XRD), Raman spectroscopy, X-ray photoelectron spectroscopy (XPS) and temperature-programmed reduction with hydrogen and methane. The performance was negatively influenced by a strong cobalt-support interaction, which in turn reduced the amount of active cobalt species as Co_3_O_4_. Hence, when alumina or magnesia supports were employed, the formation of CoAl_2_O_4_ or Co–Mg mixed oxides, respectively, with a low reducibility was evident, while ceria showed a lower affinity for deposited cobalt and this remained essentially as Co_3_O_4_. Furthermore, the observed partial insertion of Ce into the Co_3_O_4_ lattice played a beneficial role in promoting the oxygen mobility at low temperatures and consequently the catalytic activity. This catalyst also exhibited a good thermal stability while the presence of water vapor in the feedstream induced a partial inhibition, which was found to be completely reversible.

## 1. Introduction

Compressed natural gas is regarded as a suitable alternative to substitute the traditional automotive fuels such as gasoline or diesel that are becoming more expensive and scarce with time. Natural gas vehicles have been demonstrated to produce less CO_2_, NO_x_ and soot emissions than gasoline or diesel vehicles and are safer in case of accident [1,2]. Nevertheless, the application of this technology is accompanied by the necessity of controlling the emissions of unburned methane from the engine, as this is a powerful greenhouse effect gas. The most commonly applied solution is the complete oxidation over a supported noble metal catalyst, such as platinum and/or palladium [3,4]. However, the price of these metals is extremely high and they are also prone to deactivation by sintering and the presence of water, and thus, this raises the cost of natural gas engines and limits their massive implementation. For this reason, the interest in developing noble-metal free catalysts for methane oxidation is increasing.

Cobalt oxide-based catalysts, and among them spinel-type cobalt oxide (Co_3_O_4_), are considered good alternative candidates to noble metals due to their already demonstrated high efficiency for the oxidation of hydrocarbons and a greater availability [5,6,7]. The reason for this high activity seems to lie on their good redox properties such as reducibility and mobility of oxygen species at low temperatures, which derives from the easiness that the constituent ions of these materials have to shift between oxidation states. Moreover, these catalysts are more thermally stable than noble-metal based catalysts and generally more resistant to water inhibition [8,9]. However, cobalt oxides tend to present poor structural and textural properties as well, especially when they are prepared by common synthesis methods such as precipitation, sol–gel or solution combustion [10,11]. For this reason, these materials are usually supported over porous materials as a way to improve their properties, and also as a way to facilitate their incorporation into the monolithic systems where they should eventually operate [12,13].

The selection of an appropriate support is not a trivial task and can have a significant effect on the properties of the final catalyst, due to the different nature of the cobalt-support interactions, to the point of even rendering the catalyst useless for the specific purpose under study. For cobalt oxide catalysts the most commonly used supports are alumina [14,15], magnesia [16,17], zirconia [18,19], silica [20,21] ceria [22,23], silicon carbide [24,25], zeolites [26,27] or cordierite [28,29]. The decision of using one or other support for a specific cobalt-based catalyst is generally made on the basis of the specific catalytic properties that regulate the activity for the reaction under study.

Regarding this, many studies have dealt with the effect of specific supports on the performance of Co_3_O_4_ catalysts for different reactions. For instance, Grzybek et al. [30] found out that cobalt oxide showed very different behavior for N_2_O abatement depending on which polymorph of alumina was used as the support, and concluded that it was better to sacrifice the textural properties of the final catalyst by using low-surface α-Al_2_O_3_ instead of γ-Al_2_O_3_ with the objective of inhibiting the occurrence of cobalt-alumina interactions. This effect was also found by Solsona et al. [31], when studying the total oxidation of propane with cobalt oxide supported over alumina with low, medium and high surface area. On the other hand, Yung et al. [32] examined the oxidation of NO with cobalt catalysts supported over titania and zirconia, finding that the latter was a more suitable support for this purpose than the former. However, on a different study, Kim et al., [33] reported that high-surface ceria was a better support than titania or zirconia for the same reaction. Ceria was also found to be, as pointed out by Wyrwalski et al. [34], the most suitable support for cobalt oxide for the complete oxidation of propene. This type of studies has been also carried out for liquid phase reactions. For example, Zhang et al. [35] analyzed the influence of several supports for the degradation of organic dyes in solution. They concluded that MgO was the most suitable for this purpose due to the increase in the population of surface Co^2+^ ions induced by cobalt–magnesia interactions.

In the case of methane oxidation, it has been demonstrated that the population of Co^3+^ species in the spinel lattice is the key parameter that provides the catalyst with the good reducibility and oxygen mobility involved in the Mars–van Krevelen mechanism [36,37]. Thus, an appropriate support for this reaction will be one that enables a high dispersion of the cobalt deposited, while at the same time allows the cobalt oxide species to maintain their good redox properties as intact as possible. However, this is not always achievable, as a high dispersion of the deposited cobalt is usually accompanied by a strong cobalt-support interaction that, more often than not, ends up being detrimental for the oxygen mobility of the final catalyst [38].

A wide number of works have investigated the oxidation of methane over supported cobalt catalysts, each one focusing on a specific support, under different reaction conditions, with different degrees of success [39,40,41]. However, there are no studies about the effect that supports with a varying physico-chemical nature have on the fundamental properties of cobalt oxide-based catalysts, and the comparison of their activity under the same conditions. For this reason, in the present work, three Co_3_O_4_ catalysts supported over γ-alumina, magnesia and ceria were prepared by the same synthesis route, characterized and examined for the oxidation of methane under lean conditions, with the objective of determining the effect that the different supports have on the textural, structural and redox properties and the activity of the cobalt oxide active phase.

## 2. Materials and Methods

### 2.1. Synthesis of the Supports and Supported Cobalt Catalysts

Three different supports (γ-Al_2_O_3_, MgO and CeO_2_) were used for preparing the cobalt catalysts. The employed alumina was kindly provided by Saint-Gobain (Paris, France). This was previously thermally stabilized at 850 °C for 4 h in static air. Both MgO and CeO_2_ were prepared by precipitation with an aqueous solution of Na_2_CO_3_ (CAS 497-19-8) 1.2 M. This was slowly added to aqueous solutions of magnesium (II) nitrate hexahydrate (Mg(NO_3_)_2_ 6H_2_O, CAS 13446-18-9) or cerium (III) nitrate (Ce(NO_3_)_3_ 6H_2_O, CAS 10294-41-4), respectively, at a constant temperature of 80 °C, until the pH was 8.5 or 9.5. A similar synthesis route was followed for preparing the supported cobalt samples. Thus, for each support, 5 g of the selected support were mixed with 100 cm^3^ of a solution of Co(NO_3_)_2_·6H_2_O (CAS 10026-22-9) with adjusted concentration and then a solution of Na_2_CO_3_ 1.2 M was added dropwise at 80 °C until the pH reached 8.5. The nominal Co content of the three metal oxide catalysts was 30 wt. %. The samples were denoted as Co/Al_2_O_3_, Co/MgO and Co/CeO_2_. For comparative purposes, a bulk Co_3_O_4_ catalyst was also prepared (14 m^2^ g^−1^, 0.09 cm^3^ g^−1^ and 335 Å).

All samples were dried at 110 °C for 16 h and then calcined in static air to obtain the final supports (MgO and CeO_2_) and cobalt catalysts. The calcination protocol consisted on three heating ramps separated by 30-min isothermal steps at 125 and 300 °C: An initial ramp at 5 °C min^−1^ from room temperature to 125 °C, a second ramp at 1 °C min^−1^ up to 300 °C, and a final ramp at 5 °C min^−1^ up to 600 °C, temperature that was then kept constant for 4 h.

### 2.2. Characterization Techniques

Textural properties of the supports and catalysts were examined by N_2_ physisorption in a Micromeritics TriStar II apparatus (Micromeritics Instrument Corp. Norcross, GA, USA). The Brunauer-Emmett-Teller BET (Brunauer-Emmett-Teller) method was used to determine the specific surface area of the samples while the BJH (Barrett, Joyner and Halenda) method was applied for the estimation of the average pore size. Degassing of the samples prior to analysis was performed on a Micromeritics SmartPrep apparatus (Micromeritics Instrument Corp. Norcross, GA, USA). at 300 °C for 10 h with a N_2_ flow. The elemental composition of the cobalt catalysts was determined by wavelength dispersive X-ray fluorescence (WDXRF). Each sample was mixed with a flux agent (Spectromelt A12, Merck 111802, Darmstadt, Germany) in an approximate proportion of 20:1 and placed in an induction micro-furnace at 1200 °C to form a boron glass pearl. The pearls were analyzed under vacuum in a PANalytical AXIOS sequential WDXRF spectrometer (Malvern Panalytical Ltd, Royston, UK), equipped with a Rh tube and three different detectors (gas flow, scintillation and Xe sealed). 

Structural properties of the catalysts were determined by X-Ray diffraction and Raman spectroscopy. XRD analysis was performed on a X’PERT-PRO X-Ray diffractometer (Malvern Panalytical Ltd, Royston, UK). using Cu Kα radiation (λ = 1.5406 Å) and a Ni filter. The X-Ray source was operated at 40 kV and 40 mA of current. The diffractograms were obtained in the 2θ range of 5–80° with a step size of 0.026° and a counting time of 2.0 s. Phase identification was carried out by comparing the diffraction patterns with JCPDS (Joint Committee on Powder Diffraction Standards) database cards. Additionally, in order to perform a detailed XRD analysis over the supported cobalt catalysts a longer counting time (26.8 s) was applied. The cell size of the cobalt spinel phase was estimated by profile matching of the detailed XRD patterns using FullProf.2k software (version 6.30, Institut Laue-Langevin, Grenoble, France).

The analysis by Raman spectroscopy was carried out by using a Renishaw InVia Raman spectrometer (Renishaw, Wotton-under-Edge, Gloucestershire, UK), coupled to a Leica DMLM microscope (Wetzlar, Germany) with a spatial resolution of 2 microns. For each spectrum, 20 s were employed and five scans were accumulated with the 10% of the maximum power of a 514 nm laser (ion-argon laser, Modu-Laser, Centerville, UT, USA) in a spectral window of 150–1500 cm^−1^. X-ray photoelectron spectroscopy (XPS) analysis was performed using a SPECS system (SPECS GmbH, Berlin, Germany) equipped with a Phoibos 150 1D analyzer (SPECS GmbH, Berlin, Germany) and a DLD-monochromatic radiation source. The obtained spectra were calibrated by fixing the signal of adventitious carbon at 284.6 eV.

Temperature-programmed reduction with hydrogen (H_2_-TPR) analysis were carried out on a Micromeritics Autochem 2920 apparatus (Micromeritics Instrument Corp. Norcross, GA, USA), using a 5%H_2_/Ar mixture as the reducing gas. Each sample was subjected to a pre-treatment with a 5% O_2_/He mixture at 300 °C for 30 min prior to the analysis. All TPR experiments were performed up to 950 °C with an isothermal step of 10 min at that temperature. The water produced throughout each experiment was removed from the outlet stream using a cold trap, to avoid interference with the thermal conductivity detector. Additional information regarding the activation of methane was obtained by means of temperature programmed reaction with a 5% CH_4_/He mixture in the absence of oxygen (CH_4_-TPR) coupled to mass spectrometry (MKS Cirrus Quadrupole Mass Spectrometer, Andover, MA, USA). The experiments were conducted up to 600 °C with a heating ramp of 10 °C min^−1^ followed by an isothermal step at 600 °C for 30 min.

### 2.3. Evaluation of the Catalytic Performance

Catalytic activity was examined in a bench-scale fixed bed reactor (PID Eng&Tech S.L. Madrid, Spain) in the 300–600 °C temperature range at atmospheric pressure. In each reaction experiment, 1 g of catalyst was used (particle size of 0.25–0.3 mm). The catalyst was diluted with the same mass of inert quartz (particle size 0.5–0.8 mm) to ensure a good distribution of heat and reactants along the catalytic bed. The feedstream (1%CH_4_, 10%O_2_ and N_2_ as the balance gas) was fed to the reactor with a total flow of 500 cm^3^ min^−1^, which corresponded to a space velocity of 300 cm^3^ CH_4_ g^−1^ h^−1^ (60,000 h^−1^ approximately for an estimated catalyst density of 2 g cm^−3^). The temperature of the reactor was increased in a stepwise progression, with heating ramps of 1 °C min^−1^ followed by 15-min isothermal periods each 25 °C, where methane conversion and product profiles were determined. Each chromatographic analysis was performed in triplicate. Methane conversion was calculated by the difference between inlet and outlet CH_4_ concentrations. Inlet and outlet streams were analyzed using an on-line Agilent Technologies 7890N gas chromatograph (Agilent Technologies, Santa Clara, CA, USA) equipped with a thermal conductivity detector (TCD) and two columns: for the analysis of CH_4_, O_2_, N_2_ and CO, a PLOT 5A molecular sieve column was used. For CO_2_ analysis, a PLOT U column was used. To ensure that mass or heat transfer limitations were not affecting the obtained kinetic results, the criteria for intra and extra-particle mass diffusion, heat transfer and temperature gradients were checked to be above the limits, according to the Eurokin procedure [42,43].

## 3. Results

### 3.1. Characterization of the Supports

The textural properties of the commercial γ-alumina support and the as-prepared magnesia and ceria supports in terms of BET surface area, pore volume and mean pore diameter are shown in Table 1. Notable differences were noticed among the investigated supports. Hence, the commercial alumina showed the largest specific surface area (136 m^2^ g^−1^), followed by MgO (80 m^2^ g^−1^) and CeO_2_ (8 m^2^ g^−1^). This decreasing order was also consistent for the estimated pore volume of the samples. It varied from 0.55 cm^3^ g^−1^ (γ-alumina) to 0.08 cm^3^ g^−1^ (ceria). The samples showed type IV isotherms with H2 hysteresis loops.

**Figure 1:**
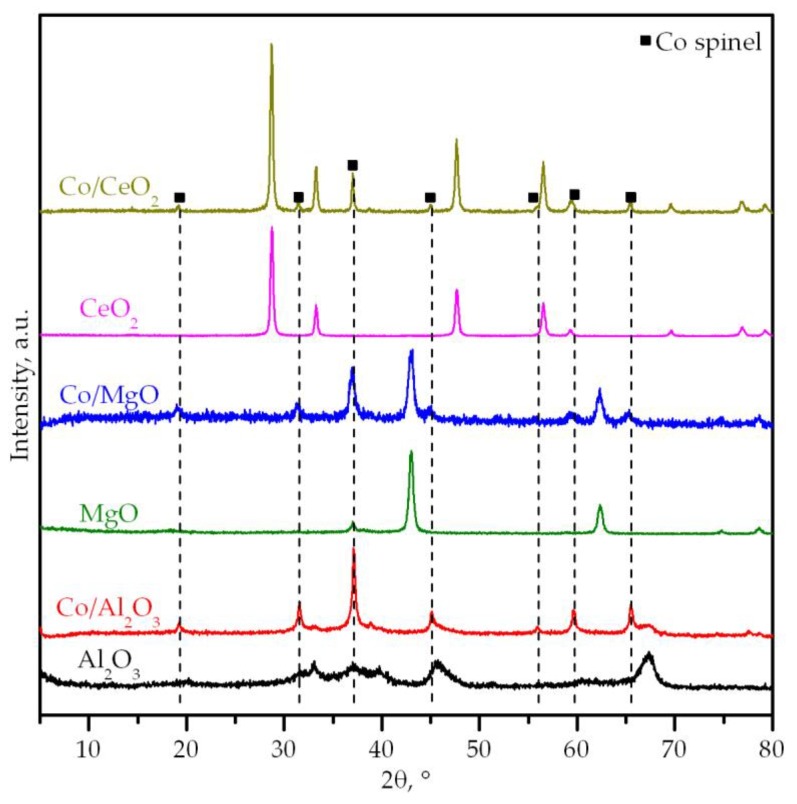
XRD patterns of the bare supports and the supported cobalt catalysts.

On the other hand, XRD patterns of the as-prepared supports well matched with those expected for the pure materials (Figure 1). Hence, the observed diffraction signals could be indexed as γ-alumina (2θ at 37.7, 45.8 and 67.3°, JCPDS 01-074-2206), magnesium oxide (2θ at 43.0, 62.3, 74.7 and 78.6°, JCPDS 00-004-0829) and cerium oxide (2θ at 28.5, 33.3, 47.5, 56.4 and 76.7°, JCPDS 00-004-0593). Moreover, the crystallinity of both magnesia and ceria was higher than that of alumina in view of their noticeably more intense and sharper signals. The formation of ceria was further corroborated by Raman spectroscopy, which revealed a strong peak assigned to the F_2g_ Raman-active mode characteristic of the fluorite-like lattice of CeO_2_. Note that the vibrational modes of MgO and γ-Al_2_O_3_ are essentially Raman inactive.

### 3.2. Characterization of the Supported Cobalt Catalysts

Table 1 lists the cobalt loading of the synthesized cobalt catalysts as determined by WDXRF and ICP-AES (Inductively Coupled Plasma-Atomic Emission Spectroscopy) in the case of the Co/CeO_2_ sample. It was verified that this content was relatively close to the nominal value (30% wt. Co). BET measurements revealed that cobalt species markedly blocked the pores of the alumina and magnesia supports, as evidenced by the notable decrease in the surface area of the Co/Al_2_O_3_ and Co/MgO catalysts, from 136 to 108 m^2^ g^−1^ (26%) and 80 to 47 m^2^ g^−1^ (42%), respectively. As with the supports, the catalysts showed type IV isotherms with H2 hysteresis loops as well. Both samples presented a lower pore volume with respect to their corresponding support (0.29 and 0.16 cm^3^ g^−1^, respectively). By contrast, the impact on the textural properties on the Co/CeO_2_ was less noticeable. Thus, a slight increase in both surface area and pore volume was found (18 m^2^ g^−1^ and 0.07 cm^3^ g^−1^, respectively).

**Figure 2:**
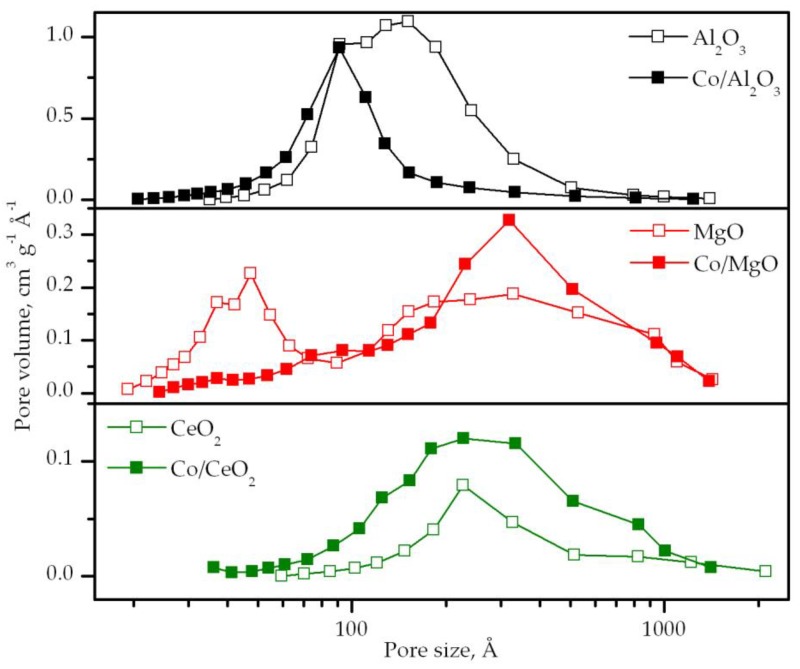
Pore size distributions of the bare supports and the supported cobalt catalysts.

The comparative analysis of the pore size distributions of the supports and the cobalt catalysts (Figure 2) evidenced that the deposition process of cobalt particles was highly dependent on the pore accessibility and interconnectivity. Hence, when cobalt was deposited over γ-alumina, which exhibited a bimodal distribution centered at 90 and 150 Å, the cobalt preferentially deposited over its largest pores. Conversely, when using magnesia as a support, characterized by pores with a markedly different size (35–50 and 325 Å), the cobalt favorably located over the smaller pores. Finally, in the case of ceria, both support and catalyst possessed a unimodal distribution centered around 225 Å, but with an increased width for the cobalt catalyst. This could be due to the fact that cobalt species did not find enough space to deposit on the pores of ceria, and subsequently located on its external surface as well. In addition, since the amount of pores of 335 Å (the prevalent pore size of bulk Co_3_O_4_) was larger in the Co/CeO_2_ catalyst than in the ceria support, it could be assumed that this catalyst contained segregated cobalt oxide to some extent.

Figure 1 includes the diffractograms of the cobalt catalysts. Their patterns were characterized by the presence of Co_3_O_4_ (2θ = 31.3, 37.0, 45.1, 59.4 and 65.3°, JCPDS 00-042-1467) along with some weak signals corresponding to the respective support (2θ = 43.0 and 62.3° and 2θ at 28.5, 47.5 and 56.4°, respectively). On the other hand, it is worth pointing out that the formation of CoAl_2_O_4_ is very frequent in Co/alumina systems due to the strong interaction between Co_3_O_4_ and the support at mild temperatures (>450 °C) [44,45]. However, from the XRD analysis the extent of the formation of this undesired phase was not possible. Note that both spinel-like cobalt phases (Co_3_O_4_ and CoAl_2_O_4_, JCPDS 00-044-0160) crystallize in the cubic structure with comparable cell parameters, thereby showing very close 2θ values in their diffraction patterns. In addition to that, the crystallinity of the support phases did not noticeably change since their crystallite size was similar before and after cobalt deposition (Table 1). However, the crystallite size of the cobalt spinel was highly dependent on the employed support. The smallest crystallite size was obtained over the magnesia (17 nm) while the largest size was found over the ceria (44 nm). This finding was consistent with the poorer textural properties of the synthesized ceria, which resulted in a preferential location of Co_3_O_4_ in its external surface. Nevertheless, in all cases the crystallite size was smaller than that of the bulk Co_3_O_4_ (63 nm), which evidenced a good dispersion of the cobalt spinel over the surface of the studied supports.

**Figure 3:**
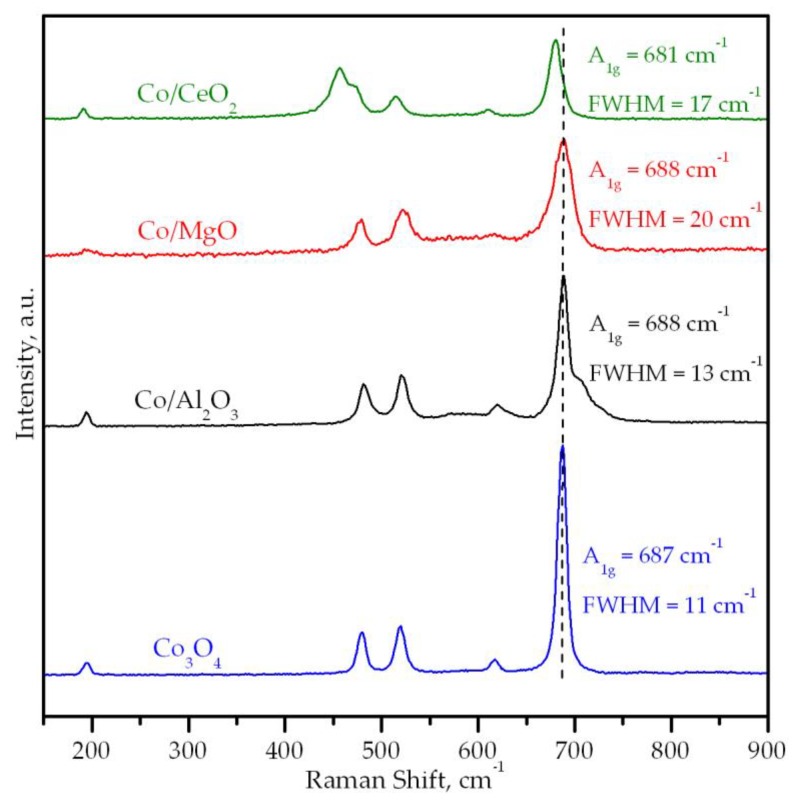
Raman spectra of the supported cobalt catalysts.

The Raman spectra in the 150–900 cm^−1^ region of the cobalt catalysts supported on alumina, magnesia and ceria are shown in Figure 3. As a reference, the spectrum of pure Co_3_O_4_ is shown as well. Apart from a relatively intense band at 462 cm^−1^ (F_2g_ mode of CeO_2_) for the Co/CeO_2_ catalyst, all supported catalysts displayed the five Raman actives modes attributable to Co_3_O_4_, namely three F_2g_ modes located at 194, 519 and 617 cm^−1^, and the E_g_ and A_1g_ modes at 479 cm^−1^ and 687 cm^−1^, respectively [46]. In addition, two shoulders at 705 and 725 cm^−1^ attached to the A_1g_ vibration mode were also visible in the case of the Co/Al_2_O_3_ catalyst. These two signals evidenced the presence of cobalt aluminate in this sample [47,48]. As far as the Co/MgO catalyst was concerned, it should be remarked that small bands at about 1250 and 1350 cm^−1^ were detected (not shown). These signals could be associated with a certain increase in the disorder of the structure of MgO owing to the insertion of cobalt ions leading to the formation of a Co–Mg solid solution [49,50]. Note that the formation of this mixed oxide was difficult to verify by XRD since no significant changes in the 2θ diffraction angles of the Co/MgO catalysts were noted with respect to those of pure MgO.

A closer inspection of the A_1g_ mode as function of the type of used support could be helpful in determining the properties of the lattice of deposited Co_3_O_4_. This influence was analyzed in terms of the shift and the full width at half maximum (FWHM) of this signal. The Raman spectra of a bulk Co_3_O_4_ were used as a reference. Thus, this Raman mode was located at 687 cm^−1^ and its FWHM was 11 cm^−1^. While no significant shift in the position of the band was found for the Co/Al_2_O_3_ (688 cm^−1^) and Co/MgO (688 cm^−1^) catalysts, a notable shift to a lower frequency (681 cm^−1^) was noted over the Co/CeO_2_ sample. This redshift of the signal could be assigned to the distortion of the spinel lattice, probably owing to insertion of Ce ions [51]. On the other hand, the largest FWHM value for the Co/MgO catalyst was assigned to the presence of Co–Mg mixed oxides. The lattice distortion in the cobalt spinel generated by the insertion of Ce ions and/or formation of Co–Mg mixed oxides was further evidenced by a slight increase in the cell parameter of the Co_3_O_4_ phase for both Co/MgO (8.083 Å) and Co/CeO_2_ (8.082 Å) catalysts with respect to the bulk Co_3_O_4_ sample (8.052 Å), where the distortion of the cobalt spinel was minimal.

**Figure 4:**
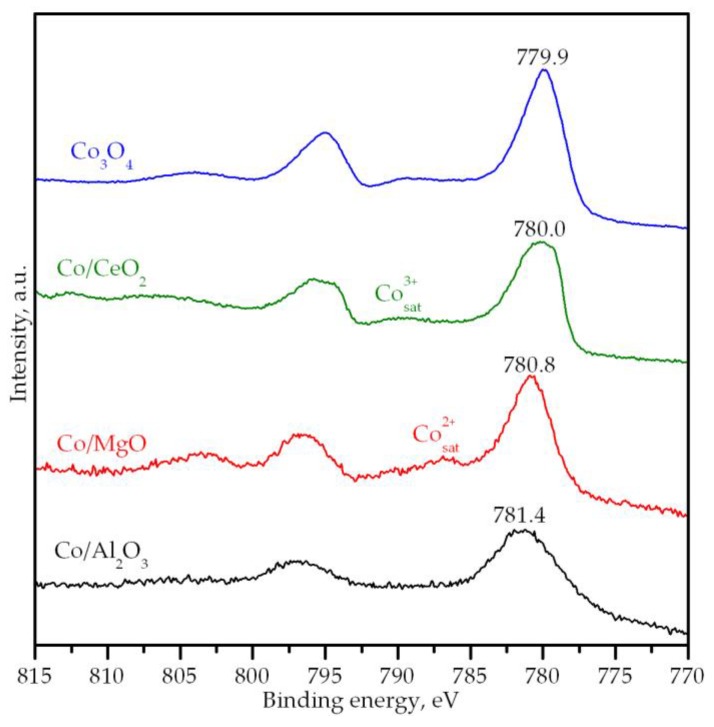
X-ray photoelectron spectroscopy (XPS) spectra of the supported cobalt catalysts.

The surface composition of the samples was investigated by XPS. The Co2p spectra of the supported cobalt catalysts are shown in Figure 4, along with the spectrum of bulk Co_3_O_4_ for the sake of comparison. All samples showed broad signals suggesting the presence of various different cobalt species on the surface of the catalysts. More specifically, all spectra showed the main Co2p_3/2_ signal in the position range 781.4–779.9 eV, along with two satellite signals centered around 785.9–786.7 eV and 789.5–790.2 eV, that were attributed to the presence of Co^2+^ and Co^3+^ ions, respectively [52]. The position of the main signal exhibited a shift with respect to the position for bulk Co_3_O_4_ (779.9 eV), which depended upon the chosen support. For the Co/CeO_2_ catalyst, the position of the main signal and the intensity of the satellite signals were comparable to that of the bulk sample. This pointed out that the nature of the cobalt oxide for this catalyst was similar to that of the bulk sample. The main signal of the Co/MgO samples was shifted towards higher binding energy values while the intensity of the Co^2+^ satellite signal was notably stronger with respect to the bulk oxide. Both features were compatible with a higher presence of Co^2+^ ions in the surface of the Co/MgO catalyst [53], as a result of the Co–Mg interaction and the subsequent formation of the Co–Mg solid solution. Lastly, for the Co/Al_2_O_3_ catalyst the main signal was located at 781.4 eV, which was a position indicative of the presence of cobalt aluminate [54]. Besides, the surface composition was determined from the integration of the XPS spectra. The respective Co/M (M = Al, Mg and Ce) surface molar ratio could be then calculated and compared with the bulk ratio calculated from the XRF analysis. For both Co/Al_2_O_3_ and Co/CeO_2_ catalysts their surface ratio (0.43 and 3.15, respectively) was higher than the bulk ratio (0.39 and 1.39, respectively), which indicated a more pronounced presence of cobalt on the surface of these samples. For the Co/MgO, however, the surface ratio (0.12) was notably lower than the bulk ratio (0.38). This could be due to the strong Co–Mg interaction and the partial insertion or dissolution of Co ions in the MgO lattice to form a Co–Mg solid solution, which in turn decreased the amount of cobalt present on the surface of this catalyst.

**Figure 5:**
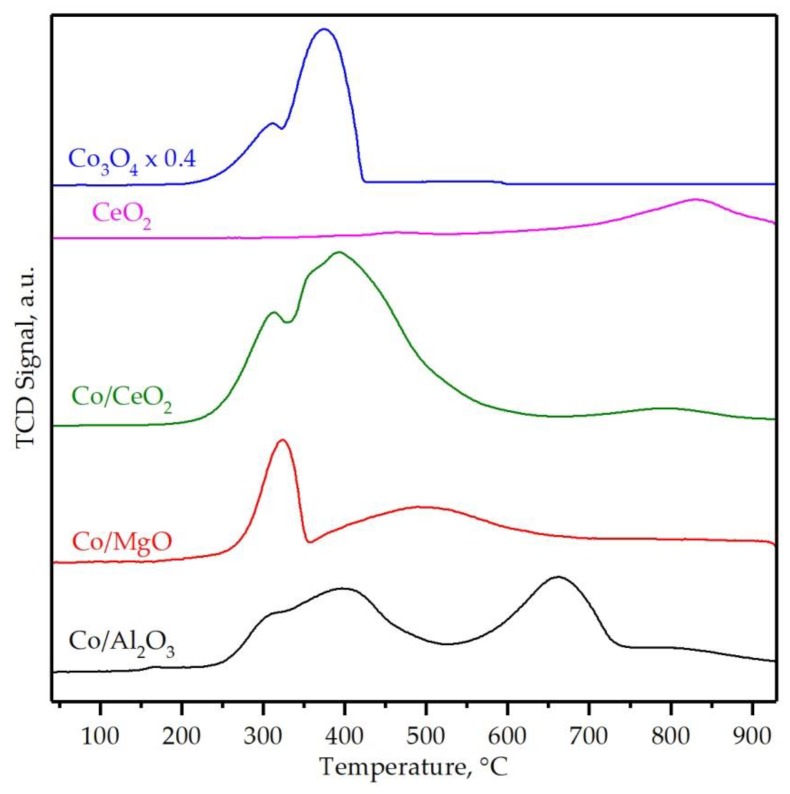
Temperature-programmed reduction with hydrogen (H_2_-TPR) profiles of the supported cobalt catalysts.

The redox properties of the catalysts were studied by temperature-programmed reduction with hydrogen in the 50–950 °C temperature range. The corresponding profiles are displayed in Figure 5 while the quantitative results of the analysis are listed in Table 2. A noticeably different redox behavior was found among the three different cobalt catalysts. Hence, the largest H_2_ uptake was exhibited by the sample supported on ceria (7.6 mmol g^−1^) followed by the catalysts supported on alumina (5.6 mmol g^−1^) and magnesia (4.6 mmol g^−1^). First, the redox properties of the latter two samples will be comparatively discussed since both γ-Al_2_O_3_ and MgO could be considered non-reducible in the studied temperature window. Hence, the observed H_2_ consumption could be exclusively assigned to the reduction of the present cobalt species. In this sense, and taking as a reference the ideal specific H_2_ uptake for the reduction of Co_3_O_4_ as the only cobalt phase (22.6 mmol H_2_ g_Co_^−1^), both Co/Al_2_O_3_ and Co/MgO catalysts revealed a significantly lower consumption, namely 18.7 and 14.5 mmol H_2_ g_Co_^−1^, respectively. Therefore, the estimated degrees of Co reduction were 88 and 64%. In line with the results given by Raman spectroscopy, these findings suggested that a fraction of deposited cobalt species strongly interacted with these supports, thereby negatively influencing their redox properties.

The reduction process of the Co/Al_2_O_3_ catalyst consisted on two main reduction events. The first event, centered at about 250–550 °C, could be attributed to the reduction of free Co_3_O_4_. This reduction process could be subsequently divided into other two features with peak reduction temperatures at 310 and 400 °C, following the same reduction process as for the bulk Co_3_O_4_ catalysts. This consisted of the sequential reduction to CoO and metallic Co, respectively [55,56]. An additional peak between 550–750 °C was clearly ascertained. This was attributed to the presence of significant amounts of CoAl_2_O_4_ derived from the strong interaction between Co_3_O_4_ and Al_2_O_3_. It was quantitatively deduced that the total amount of cobalt of the sample was equally distributed as Co_3_O_4_ (49%) and CoAl_2_O_4_ (51%). It must be noted that all cobalt species present in the catalysts were completely reduced to metallic Co. This was verified by XRD analysis of the samples recovered after the TPR run where only metallic Co and Al_2_O_3_ were detected.

The H_2_-TPR profile of the Co/MgO sample also revealed two distinct reduction regions although the temperatures windows were markedly different when compared with the alumina-supported counterpart. Hence, the observed consumption at low-temperature (200–350 °C) was ascribed to easily reducible free Co_3_O_4_ species as well. The band located at higher temperatures (350–650 °C) was assigned to the reduction of cobalt-MgO species formed during the synthesis route. The integration of these two features gave the following cobalt distribution, 57% as Co_3_O_4_ and 43% as Co–Mg mixed oxides. The formation of Co–Mg mixed oxides with a superior stability, which could not be reduced even at 950 °C, could not be however ruled out, as the total H_2_ uptake of this sample was rather low in view of its Co content [17,57]. In fact, when assuming that the uptake at low temperatures (<350 °C) was owing to the reduction of Co_3_O_4_ with a stoichiometry of 4 moles of H_2_:3 moles of Co, and the uptake at higher temperatures (350–650 °C) was related to the reduction of Co^2+^ species with a stoichiometry of 1 mol of H_2_: 1 mol of Co, the corresponding metal content of the sample, in view of its overall H_2_ uptake, would be equivalent to about 25% wt. This was considerable lower than the actual Co loading as determined by XRF (close to 32% wt.). Anyway, the amount of free Co_3_O_4_, with a significantly higher oxidation activity in comparison with CoAl_2_O_4_ or Co-Mg mixed oxides, was relatively similar for both γ-Al_2_O_3_ and MgO supported catalysts.

While the H_2_ uptake of both pure alumina and magnesia was negligible, the bare CeO_2_ sample exhibited a weak signal at 450–500 °C that corresponded to the surface reduction of the oxide whereas the notable H_2_ consumption peaking at about 825 °C was related to the reduction of the bulk [58,59]. The TPR profile of the Co/CeO_2_ catalyst was characterized by a remarkable uptake between 200–600 °C that was related to the reduction of precipitated Co_3_O_4_. Similarly to the Co/Al_2_O_3_ sample, this feature exhibited two fairly discernible peaks at 310 and 380 °C. A small band was noted at 800 °C as well, which corresponded to the reduction of the bulk of the support. Note that this occurred to slightly lower temperatures with respect to the bare support, probably due to the catalytic role played by cobalt. A quantitative analysis of the amount of consumed H_2_ revealed that the overall uptake (7.6 mmol H_2_ g^−1^) reasonably matched with that theoretically expected for the total reduction of Co_3_O_4_ along with the reduction of ceria (1.8 mmol H_2_ g_Ce_^−1^). This corresponded to a 100% degree of Co reduction (Table 2). At low temperatures, the amount of consumed H_2_ was 23.4 mmol g_Co_^−1^, which was slightly larger than theoretically required for the reduction of the Co_3_O_4_ oxide (22.6 mmol g_Co_^−1^). This suggested that deposited cobalt facilitated the reduction of the surface of the ceria in this temperature range [60].

An overall overview of the redox properties of the three cobalt catalysts pointed out that the use of ceria was beneficial for obtaining a sample with a limited interaction of the active phase with the surface support, thereby not favoring the formation of hardly reducible cobalt oxides such as cobalt aluminate or cobalt–magnesium mixed oxides. In addition, cobalt helped in promoting the reducibility of the ceria.

**Figure 6:**
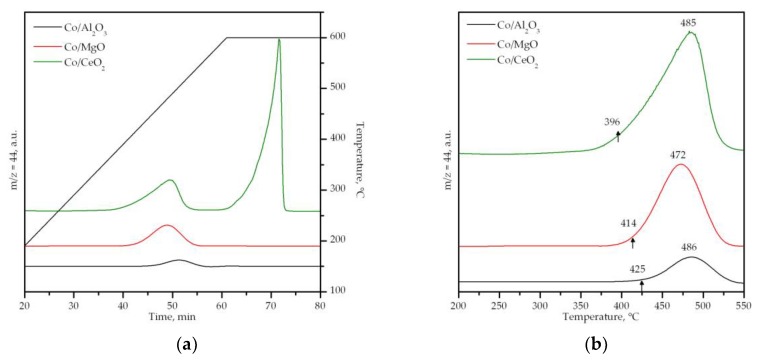
Profiles of the supported cobalt catalysts (**a**). Close-up view of the 200–550 °C temperature range (**b**).

More useful insights on the influence of the catalyst composition on the reactivity of available active oxygen species for methane oxidation were obtained by CH_4_-TPR analysis coupled to mass spectrometry. The analysis was performed between 50 and 600 °C with a subsequent isothermal step at this temperature for 30 minutes. The evolution of CO_2_ (m/z = 44) and CO (m/z = 28, not shown) was monitored (Figure 6). In the low temperature range, namely 375–525 °C, the generation of CO_2_ was noticed over the three cobalt catalysts (peaking at about 485 °C). This was attributed to the oxidation of methane by oxygen species associated with Co^3+^ ions. However, the extent of this reaction was considerably different over each sample in view of the comparatively larger amount of consumed oxygen (0.70 mmol O_2_ g^−1^) or larger yield of CO_2_ over the Co/CeO_2_ sample, followed by the Co/MgO (0.51 mmol O_2_ g^−1^) and Co/Al_2_O_3_ (0.28 mmol O_2_ g^−1^) catalysts. (Table 2) Moreover, the temperature for the onset of reduction (marked by arrows in Figure 6) was significantly lower for the Co/CeO_2_ sample (395 °C) in comparison with the other two catalysts, namely 415 and 425 °C over Co/MgO and Co/Al_2_O_3_, respectively.

On the other hand, during the isothermal period at 600 °C a second oxidation process was only evidenced over the Co/CeO_2_ sample. This was also accompanied by the generation of CO and H_2_ to some extent that was related to partial oxidation or cracking of methane in the presence of metallic or oxygen-deficient cobalt species [61]. In fact, the diffraction pattern of the sample after the CH_4_-TPR run evidenced the formation of graphitic carbon (signal at 2θ = 26.6°) [62]. The distinct formation at high temperatures of these species (CO_2_, CO and H_2_) was not observed over the other two catalysts, thereby revealing the presence of very stable, inactive cobalt species on these samples, such as cobalt aluminate or cobalt–magnesium mixed oxides, where cobalt was mainly present as Co^2+^.

### 3.3. Performance of the Supported Cobalt Catalysts

**Figure 7:**
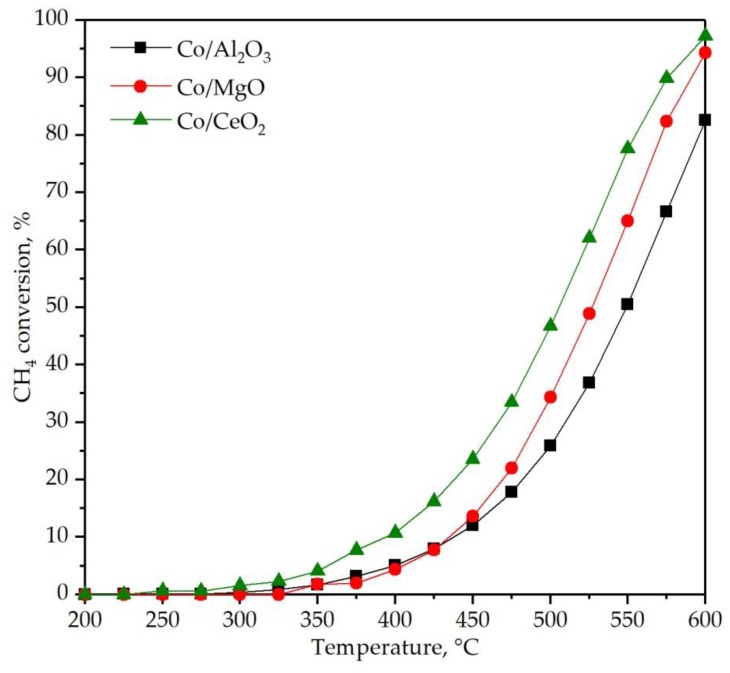
Light-off curves of the supported cobalt catalysts.

The performance of the cobalt catalysts was examined by their corresponding light-off curves at 300 cm^3^ CH_4_ g^−1^ h^−1^ (30,000 cm^3^ g^−1^ h^−1^, about 60,000 h^−1^) in the 200–600 °C temperature range. For each catalyst, three consecutive reaction cycles were conducted. In all cases the first light-off curve revealed slightly lower reaction temperatures while the second and third runs were identical to each other, as can be seen for the Co/CeO_2_ catalyst in Figure S1 (Supplementary Materials). For this reason, Figure 7 compares the 3rd-cycle curve for each examined catalyst. Recall that only CO_2_ was detected in the product stream in the whole temperature range. Hence, a 100% selectivity towards CO_2_ formation was achieved for all tested catalysts. Appreciable methane conversion (>5%) was detected over 350 °C over the Co/CeO_2_ catalysts while a similar conversion level was attained at significantly higher temperatures (400 °C) over the samples supported on both magnesia and alumina. The T_50_ value (temperature at which 50% conversion was attained) was used as criterion for the relative reactivity of each sample (Table 3). In a similar way to the results observed in the low-conversion range, a substantially different performance was addressed with values close to 500 °C (Co/CeO_2_), 525 °C (Co/MgO) and 550 °C (Co/Al_2_O_3_). Accordingly, conversion values at around 85% (Co/Al_2_O_3_), 95% (Co/MgO) and 98% (Co/CeO_2_) were noted at 600 °C.

An additional evidence of the goodness of the Co/CeO_2_ sample was given by the analysis of the specific reaction rate of the cobalt catalysts (Table 3). This reaction rate was calculated under differential conditions (conversion < 20%) at 425 °C. The ceria supported sample exhibited a markedly higher specific activity (3.1 mmol CH_4_ g_Co_^−1^ h^−1^) with respect to the other two counterparts, which showed a similar performance (1.4–1.8 mmol CH_4_ g_Co_^−1^ h^−1^).

**Figure 8:**
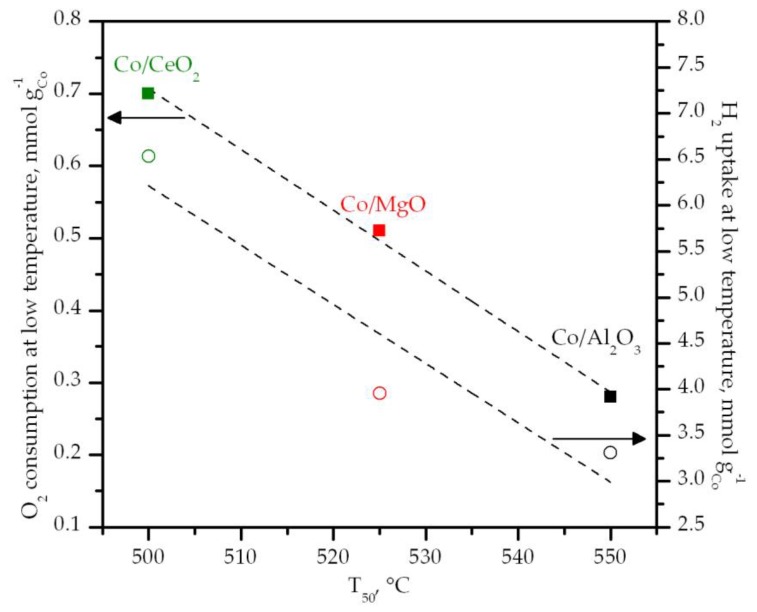
Relationship between the activity and the redox properties of the supported catalysts.

On the basis of the fact that methane oxidative decomposition over cobalt catalysts requires highly active oxygen species [63,64], the observed trend in catalytic activity was proposed to be directly related to the amount of easily reducible cobalt species in each sample, which could be measured by the specific oxygen consumption at low temperatures in the CH_4_-TPR profiles. In this sense, Figure 8 shows that there was a markedly good correlation between the T_50_ values and the reacted O_2_ below 550 °C. Accordingly, a comparable relationship was evidenced in relation with the H_2_ uptake involved in the reduction of the free Co_3_O_4_ present in each supported cobalt catalyst (H_2_-TPR profiles).

**Figure 9:**
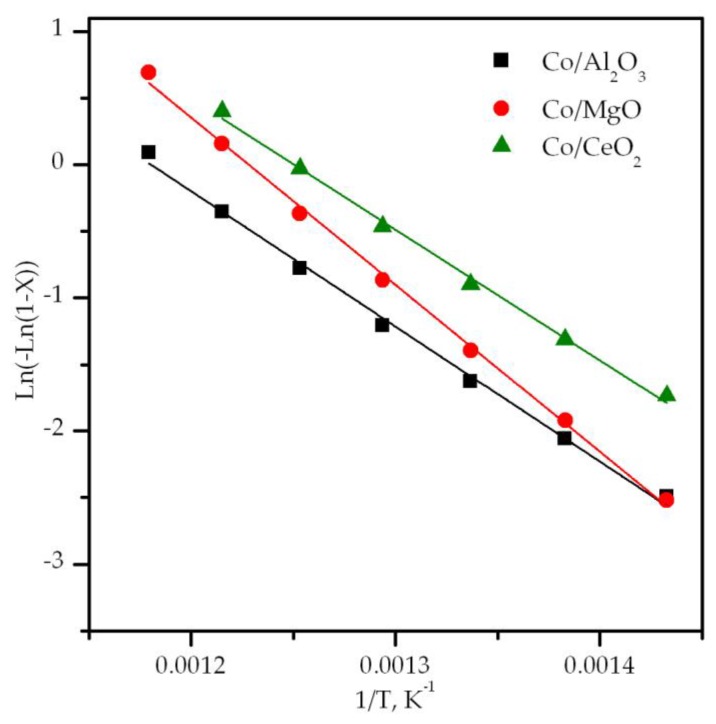
Order integral fit for the experimental kinetic data obtained over the supported cobalt catalysts.

The integral method was followed for evaluating the apparent activation energy of the reaction over the examined cobalt catalysts. A power law kinetic equation, derived from a simplified Mars–van Krevelen reaction mechanism in excess of oxygen, was used. Hence, methane was assumed to follow first pseudo-order kinetics while for oxygen a zero pseudo-order was used [65]. The results are listed in Table 3 while the corresponding plots for this linearized kinetic equation are shown in Figure 9. In addition, the activation enthalpy and entropy (Table 3) were estimated by applying the Eyring-Polanyi equation. The corresponding linearized plots are shown in Figure S2 (Supplementary Materials). The following apparent activation energies were estimated, namely 82 kJ mol^−1^ over Co/CeO_2_, 90 kJ mol^−1^ over Co/Al_2_O_3_ and 102 kJ mol^−1^ over Co/MgO. When compared with the value obtained by the bulk Co_3_O_4_ (78 kJ mol^−1^) a close similarity was found in relation to the Co/CeO_2_ catalyst. This finding was coherent with the fact that in both cases the nature of the active cobalt phase was the same, namely, Co_3_O_4_. A noticeable higher activation energy was noticed for the samples in which a mixture of cobalt phases was present, namely Co_3_O_4_/CoAl_2_O_4_ (Co/Al_2_O_3_ catalyst) and Co_3_O_4_/Co-Mg mixed oxide (Co/MgO catalyst). This behavior could lie in the contribution of these intrinsically less active phases to the reaction mechanism, especially in the case of the Co/MgO given its different activation entropy, thereby negatively influencing the overall activity of the resultant catalyst [57,66].

In this way, it could be established that the presence of CoAl_2_O_4_ would negatively affect the intrinsic activity of the Co/Al_2_O_3_ samples while the formation of a stable Co–Mg solid solution would negatively impact on the kinetic behavior of the Co/MgO catalyst.

**Figure 10:**
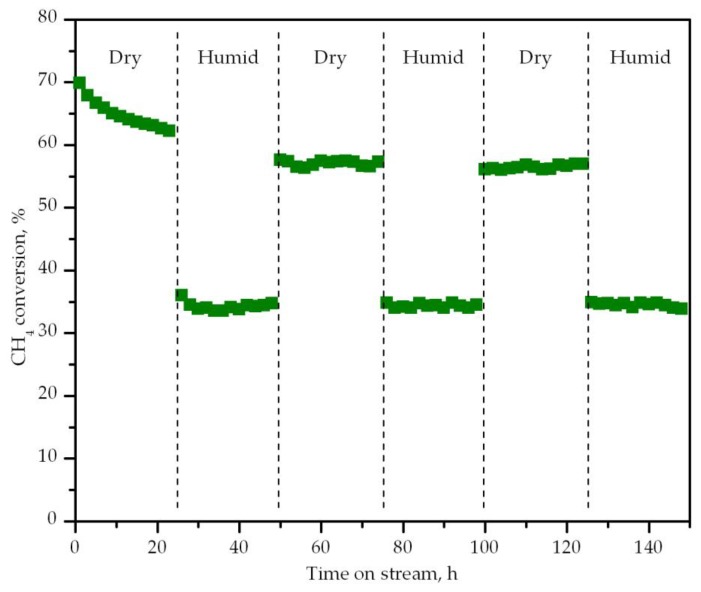
Stability of the Co/CeO_2_ catalyst under cycled dry/humid conditions with time on stream.

Finally, the thermal and hydrothermal stability of the most active catalyst (Co/CeO_2_) was studied over relatively prolonged periods of operation under both dry and humid conditions at 300 cm^3^ CH_4_ g^−1^ h^−1^ (30,000 cm^3^ g^−1^ h^−1^, about 60,000 h^−1^). A 1% CH_4_/10% O_2_/10% H_2_O/79% N_2_ was used for the time intervals carried out in the presence of water. The dry and humid conditions where switched every 25 h over a total operation span of 150 h at a constant temperature of 550 °C. The results are shown in Figure 10. The catalyst underwent some fast thermal deactivation during the first 25 h of the test where the conversion dropped from 70% to 60%. The introduction of water to the feed stream had a significantly detrimental effect over the activity of the catalyst, and as a result conversion levels fell down to about 35%. However, when the dry conditions were re-established, the conversion recovered almost completely to the levels exhibited before the addition of water. This behavior was again seen during the following dry-humid operation cycles, and suggested that water inhibition was essentially caused by the coverage of the surface that limited the extent of the reaction methane and catalyst oxygen active species. This effect has been also observed in other works, and has been generally been linked to the weak adsorption potential that water molecules have on the surface of cobalt oxide [67,68]. In particular, Geng et al. [69] observed a significant increase in the intensity of the DRIFTS absorption bands from hydroxyl groups in a Co/γ-Al_2_O_3_ catalyst when water vapor was added to the feed. Additionally, H_2_O-TPD experiments in that work also proved that a high fraction of adsorbed water on the Co catalyst surface could be desorbed at low temperatures (<500 °C).

## 4. Conclusions

Cobalt catalysts supported over gamma-alumina, magnesia and ceria were synthesized, characterized and examined for the oxidation of methane under lean conditions with the objective of determining the influence of the catalytic support on the properties and activity of the active Co_3_O_4_ phase.

The analysis of the samples evidenced a marked effect of the strong cobalt-support interaction on the catalytic performance. More specifically, when the selected support was γ-alumina, the high cobalt dispersion and strong cobalt-support interaction resulted in the partial fixation of deposited cobalt as poorly reducible cobalt aluminate (CoAl_2_O_4_) species. This led to a low activity despite the good structural and textural properties of the resultant catalyst. In the case of the Co/MgO sample, the cobalt-support interaction provoked the partial dissolution and insertion of cobalt cations into the MgO lattice with the subsequent formation of a highly stable Co–Mg solid solution. However, this catalyst was somewhat more active than the alumina counterpart, due to a higher oxygen mobility of the remaining free Co_3_O_4_ phase. Lastly, when the chosen support was ceria, the resultant catalyst exhibited worse structural and textural properties, but interestingly a noticeably promoted reducibility and oxygen mobility caused by the partial insertion of Ce cations into the cobalt spinel lattice. These beneficial effects made this catalyst the most active sample among the examined cobalt catalysts. Additionally, while its thermal stability over a prolonged time interval was found to be high, the addition of water vapor to the feedstream provoked a reversible inhibition assigned to the coverage of the catalyst surface by water molecules.

Given the good activity showed by the Co/CeO_2_ catalyst in spite of its poor textural and structural properties, future efforts will be focused on designing Co/CeO_2_ with improved specific surface area and crystallite size, either by modification of the synthesis method of the ceria support or by selecting ceria as a promoter for Co/CeO_2_–Al_2_O_3_ catalysts.

## Figures and Tables

**Table 1:** Textural properties of the supports and the supported cobalt catalysts.

Sample	Surface Area, m^2^ g^−1^	Pore Volume, cm^3^ g^−1^	Mean Pore Diameter, Å	Cobalt Content, wt.%	Support Crystallite Size, nm	Co_3_O_4_ Crystallite Size, nm	Co/M Molar Ratio^1^
Al_2_O_3_	136	0.55	123	-	5	-	-
MgO	80	0.19	106	-	21	-	-
CeO_2_	8	0.03	230	-	32	-	-
Co/Al_2_O_3_	108	0.29	89	27.9	6	29^2^	0.43 (0.39)
Co/MgO	47	0.16	204	31.9	16	17	0.12 (0.38)
Co/CeO_2_	18	0.07	225	28.9	33	44	3.15 (1.39)

^1^ The values in brackets correspond to the molar ratio determined by X-ray fluorescence (XRF). M stands for metal of the oxide support (Al, Mg and Ce, respectively). ^2^ This value corresponds to the size of the spinel Co_3_O_4_–CoAl_2_O_4_ phases present in this sample, which are undistinguishable by XRD.

**Table 2:** Redox properties of the supported cobalt catalysts derived from TPR analysis with H_2_ and CH_4_.

Sample	Total H_2_ Uptake^1^ mmol g_Co_^−1^	H_2_ Uptake at Low Temperature^1^ mmol g_Co_^−1^	Degree of Co Reduction^2^ %	O_2_ Consumption at Low Temperature (CH_4_-TPR) mmol g_Co_^−1^
Co/Al_2_O_3_	18.7 (5.6)	9.8 (2.7)	88	0.28
Co/MgO	14.5 (4.6)	9.9 (3.2)	64	0.51
Co/CeO_2_	26.2 (7.6)	23.4 (6.8)	100	0.70
CeO_2_	- (1.5)	-	-	-

^1^ The values in brackets correspond to the H_2_ uptake on a catalyst weight basis. ^2^ This degree of reduction was estimated based on 22.6 mmol H_2_ g_Co_^−1^ for full reduction of cobalt as Co_3_O_4_.

**Table 3:** Kinetic results of the oxidation of lean methane over the supported cobalt catalysts.

Sample	T_50_ °C	Specific Rate at 425 °C mmol CH_4_ g_Co_^−1^ h^−1^	E_a_ kJ mol^−1^	ΔH^‡^ kJ mol^−1^	ΔS^‡^ J mol^−1^ K^−1^
Co/Al_2_O_3_	550	1.8	90	84	−166
Co/MgO	525	1.4	102	98	−139
Co/CeO_2_	500	3.1	82	78	−161

## Supplementary Materials

The following are available online at https://www.mdpi.com/1996-1944/12/19/3174/s1.

## References

[1] Yuan Z., Ou X., Peng T., Yan X. (2019). Life cycle greenhouse gas emissions of multi-pathways natural gas vehicles in china considering methane leakage. Appl. Energy.

[2] Vinoth Kanna I., Arulprakasajothi M., Eliyas S. (2019). A detailed study of IC engines and a novel discussion with comprehensive view of alternative fuels used in petrol and diesel engines. Int. J. Ambient Energy.

[3] Rink M., Eigenberger G., Nieken U. (2013). Comparison of two different heat-integrated exhaust purification devices for monovalent CNG engines. Top. Catal..

[4] Kim J., Kim E., Han J., Han H.S. (2013). Pt/Pd bimetallic catalyst with improved activity and durability for lean-burn CNG engines. SAE Int. J. Fuels Lubr..

[5] Cai T., Huang H., Deng W., Dai Q., Liu W., Wang X. (2015). Catalytic combustion of 1,2-dichlorobenzene at low temperature over Mn-modified Co_3_O_4_ catalysts. Appl. Catal. B Environ..

[6] Bai B., Arandiyan H., Li J. (2013). Comparison of the performance for oxidation of formaldehyde on nano-Co_3_O_4_, 2D-Co_3_O_4_, and 3D-Co_3_O_4_ catalysts. Appl. Catal. B Environ..

[7] Tian Z., Tchoua Ngamou P.H., Vannier V., Kohse-Höinghaus K., Bahlawane N. (2012). Catalytic oxidation of VOCs over mixed Co-Mn oxides. Appl. Catal. B Environ..

[8] Chen Z., Wang S., Liu W., Gao X., Gao D., Wang M., Wang S. (2016). Morphology-dependent performance of Co_3_O_4_ via facile and controllable synthesis for methane combustion. Appl. Catal. A Gen..

[9] Setiawan A., Kennedy E.M., Dlugogorski B.Z., Adesina A.A., Stockenhuber M. (2015). The stability of Co_3_O_4_, Fe_2_O_3_, Au/Co_3_O_4_ and Au/Fe_2_O_3_ catalysts in the catalytic combustion of lean methane mixtures in the presence of water. Catal. Today.

[10] Ercolino G., Stelmachowski P., Kotarba A., Specchia S. (2017). Reactivity of mixed iron–cobalt spinels in the lean methane combustion. Top. Catal..

[11] Chen J., Arandiyan H., Gao X., Li J. (2015). Recent advances in catalysts for methane combustion. Catal. Surv. Asia.

[12] Wójcik S., Grzybek G., Gryboś J., Kotarba A., Sojka Z. (2018). Designing, optimization and performance evaluation of the K-Zn_0.4_Co_2.6_O_4_|α-Al_2_O_3_|cordierite catalyst for low-temperature N_2_O decomposition. Catal. Commun..

[13] Wang Q., Peng Y., Fu J., Kyzas G.Z., Billah S.M.R., An S. (2015). Synthesis, characterization, and catalytic evaluation of Co_3_O_4_/γ-Al_2_O_3_ as methane combustion catalysts: Significance of Co species and the redox cycle. Appl. Catal. B Environ..

[14] Li Y., Wang X. (2019). MgO modifying Al_2_O_3_ to load cobalt oxide for catalytic N_2_O decomposition. Catal. Lett..

[15] Zacharaki I., Kontoyannis C.G., Boghosian S., Lycourghiotis A., Kordulis C. (2009). Cobalt oxide supported on alumina catalysts prepared by various methods for use in catalytic afterburner of PEM fuel cell. Catal. Today.

[16] Kobayashi Y., Iwasaki Y. (2017). Fabrication of Macroporous Co_3_O_4_–MgO composite catalysts for methylene blue degradation using ozone as an oxidant. J. Chem. Eng. Jpn..

[17] Ulla M.A., Spretz R., Lombardo E., Daniell W., Knözinger H. (2001). Catalytic combustion of methane on Co/MgO: Characterisation of active cobalt sites. Appl. Catal. B Environ..

[18] Ercolino G., Stelmachowski P., Specchia S. (2017). Catalytic performance of Pd/Co_3_O_4_ on SiC and ZrO_2_ open cell foams for process intensification of methane combustion in lean conditions. Ind. Eng. Chem. Res..

[19] Bai L., Wyrwalski F., Safariamin M., Bleta R., Lamonier J., Przybylski C., Monflier E., Ponchel A. (2016). Cyclodextrin-cobalt (II) molecule-ion pairs as precursors to active Co_3_O_4_/ZrO_2_ catalysts for the complete oxidation of formaldehyde: Influence of the cobalt source. J. Catal..

[20] Pudukudy M., Yaakob Z. (2015). Methane decomposition over Ni, Co and Fe based monometallic catalysts supported on sol gel derived SiO_2_ microflakes. Chem. Eng. J..

[21] Jozwiak W.K., Szubiakiewicz E., Góralski J., Klonkowski A., Paryjczak T. (2004). Physico-chemical and catalytic study of the Co/SiO_2_ catalysts. Kinet. Catal..

[22] Grzybek G., Stelmachowski P., Gudyka S., Indyka P., Sojka Z., Guillén-Hurtado N., Rico-Pérez V., Bueno-López A., Kotarba A. (2016). Strong dispersion effect of cobalt spinel active phase spread over ceria for catalytic N_2_O decomposition: The role of the interface periphery. Appl. Catal. B Environ..

[23] Liotta L.F., Di Carlo G., Pantaleo G., Deganello G. (2007). Catalytic performance of Co_3_O_4_/CeO_2_ and Co_3_O_4_/CeO_2_–ZrO_2_ composite oxides for methane combustion: Influence of catalyst pretreatment temperature and oxygen concentration in the reaction mixture. Appl. Catal. B Environ..

[24] Klegova A., Inayat A., Indyka P., Gryboś J., Sojka Z., Pacultová K., Schwieger W., Volodarskaja A., Kuśtrowski P., Rokicińska A. (2019). Cobalt mixed oxides deposited on the SiC open-cell foams for nitrous oxide decomposition. Appl. Catal. B Environ..

[25] Song H., Zhao Q., Zhou X., Cao Z., Luo M. (2018). Selection of highly active and stable Co supported SiC catalyst for Fischer-Tropsch synthesis: Effect of the preparation method. Fuel.

[26] Zhu Z., Lu G., Zhang Z., Guo Y., Guo Y., Wang Y. (2013). Highly active and stable Co_3_O_4_/ZSM-5 catalyst for propane oxidation: Effect of the preparation method. ACS Catal..

[27] Laugel G., Arichi J., Bernhardt P., Molière M., Kiennemann A., Garin F., Louis B. (2009). Preparation and characterisation of metal oxides supported on SBA-15 as methane combustion catalysts. C. R. Chim..

[28] Jirátová K., Balabánová J., Kovanda F., Klegová A., Obalová L., Fajgar R. (2017). Cobalt oxides supported over ceria–zirconia coated cordierite monoliths as catalysts for deep oxidation of ethanol and N_2_O decomposition. Catal. Lett..

[29] Bahlawane N. (2006). Kinetics of methane combustion over CVD-made cobalt oxide catalysts. Appl. Catal. B Environ..

[30] Grzybek G., Ciura K., Wójcik S., Gryboś J., Indyka P., Inger M., Antoniak-Jurak K., Kowalik P., Kotarba A., Sojka Z. (2017). On the selection of the best polymorph of Al_2_O_3_ carriers for supported cobalt nano-spinel catalysts for N_2_O abatement: An interplay between preferable surface spreading and damaging active phase-support interaction. Catal. Sci. Technol..

[31] Solsona B., Davies T.E., Garcia T., Vázquez I., Dejoz A., Taylor S.H. (2008). Total oxidation of propane using nanocrystalline cobalt oxide and supported cobalt oxide catalysts. Appl. Catal. B Environ..

[32] Yung M.M., Holmgreen E.M., Ozkan U.S. (2007). Cobalt-based catalysts supported on titania and zirconia for the oxidation of nitric oxide to nitrogen dioxide. J. Catal..

[33] Kim D.S., Kim Y.H., Yie J.E., Park E.D. (2010). NO oxidation over supported cobalt oxide catalysts. Korean J. Chem. Eng..

[34] Wyrwalski F., Giraudon J.-M., Lamonier J.-F. (2010). Synergistic coupling of the redox properties of supports and cobalt oxide Co_3_O_4_ for the complete oxidation of volatile organic compounds. Catal. Lett..

[35] Zhang W., Tay H.L., Lim S.S., Wang Y., Zhong Z., Xu R. (2010). Supported cobalt oxide on MgO: Highly efficient catalysts for degradation of organic dyes in dilute solutions. Appl. Catal. B Environ..

[36] Lukashuk L., Yigit N., Rameshan R., Kolar E., Teschner D., Hävecker M., Knop-Gericke A., Schlögl R., Föttinger K., Rupprechter G. (2018). Operando insights into CO oxidation on cobalt oxide catalysts by NAP-XPS, FTIR, and XRD. ACS Catal..

[37] Zasada F., Janas J., Piskorz W., Gorczynska M., Sojka Z. (2017). Total oxidation of lean methane over cobalt spinel nanocubes controlled by the self-adjusted redox state of the catalyst: Experimental and theoretical account for interplay between the Langmuir-Hinshelwood and Mars-Van Krevelen mechanisms. ACS Catal..

[38] Choya A., de Rivas B., González-Velasco J.R., Gutiérrez-Ortiz J.I., López-Fonseca R. (2018). Oxidation of residual methane from VNG vehicles over Co_3_O_4_-based catalysts: Comparison among bulk, Al_2_O_3_-supported and Ce-doped catalysts. Appl. Catal. B Environ..

[39] Dou J., Tang Y., Nie L., Andolina C.M., Zhang X., House S., Li Y., Yang J., Tao F. (2018). Complete oxidation of methane on Co_3_O_4_/CeO_2_ nanocomposite: a synergic effect. Catal. Today.

[40] Kumar M., Rattan G., Prasad R. (2017). Optimisation of cobalt loading on γ-Al_2_O_3_ for total oxidation of methane. Indian Chem. Eng..

[41] Jodłowski P.J., Kryca J., Iwaniszyn M., Jȩdrzejczyk R., Thomas J., Kołodziej A., Łojewska J. (2013). Methane combustion modelling of wire gauze reactor coated with Co_3_O_4_–CeO_2_, Co_3_O_4_–PdO catalysts. Catal. Today.

[42] EUROKIN Spreadsheet on Requirements for Measurement of Intrinsic Kinetics in The Gas-Solid Fixed-Bed Reactor. http://eurokin.org/.

[43] Aranzabal A., González-Marcos J.A., Ayastuy J.L., González-Velasco J.R. (2006). Kinetics of Pd/alumina catalysed 1,2-dichloroethane gas-phase oxidation. Chem. Eng. Sci..

[44] Li D., Ding Y., Wei X., Xiao Y., Jiang L. (2015). Cobalt-aluminum mixed oxides prepared from layered double hydroxides for the total oxidation of benzene. Appl. Catal. A Gen..

[45] Zhang L., Dong L., Yu W., Liu L., Deng Y., Liu B., Wan H., Gao F., Sun K., Dong L. (2011). Effect of cobalt precursors on the dispersion, reduction, and CO oxidation of CoO_x_/γ-Al_2_O_3_ catalysts calcined in N_2_. J. Colloid Interface Sci..

[46] Liu Q., Wang L.C., Chen M., Cao Y., He H.Y., Fan K.N. (2009). Dry citrate-precursor synthesized nanocrystalline cobalt oxide as highly active catalyst for total oxidation of propane. J. Catal..

[47] Jiang X., Ma Y., Chen Y., Li Y., Ma Q., Zhang Z., Wang C., Yang Y. (2018). Raman analysis of cobalt blue pigment in blue and white porcelain: A reassessment. Spectrochim. Acta Part. A Mol. Biomol. Spectrosc..

[48] D’Ippolito V., Andreozzi G.B., Bersani D., Lottici P.P. (2015). Raman fingerprint of chromate, aluminate and ferrite spinels. J. Raman Spectrosc..

[49] Wu M., Fu Y., Zhan W., Guo Y., Guo Y., Wang Y., Lu G. (2017). Catalytic performance of MgO-supported Co catalyst for the liquid phase oxidation of cyclohexane with molecular oxygen. Catalysts.

[50] Cazzanelli E., Kuzmin A., Mariotto G., Mironova-Ulmane N. (2003). Study of vibrational and magnetic excitations in Ni_c_Mg_1-c_O solid solutions by Raman spectroscopy. J. Phys. Condens. Matter.

[51] Zou G., Xu Y., Wang S., Chen M., Shangguan W. (2015). The synergistic effect in Co-Ce oxides for catalytic oxidation of diesel soot. Catal. Sci. Technol..

[52] Biesinger M.C., Payne B.P., Grosvenor A.P., Lau L.W.M., Gerson A.R., Smart R.S.C. (2011). Resolving surface chemical states in XPS analysis of first row transition metals, oxides and hydroxides: Cr, Mn, Fe, Co and Ni. Appl. Surf. Sci..

[53] Yang J., Liu H., Martens W.N., Frost R.L. (2010). Synthesis and characterization of cobalt hydroxide, cobalt oxyhydroxide, and cobalt oxide nanodiscs. J. Phys. Chem. C.

[54] Duan X., Pan M., Yu F., Yuan D. (2011). Synthesis, structure and optical properties of CoAl_2_O_4_ spinel nanocrystals. J. Alloy. Compd..

[55] Deng J., Zhang L., Dai H., Xia Y., Jiang H., Zhang H., He H. (2010). Ultrasound-assisted nanocasting fabrication of ordered mesoporous MnO_2_ and Co_3_O_4_ with high surface areas and polycrystalline walls. J. Phys. Chem. C.

[56] Liotta L.F., Wu H., Pantaleo G., Venezia A.M. (2013). Co_3_O_4_ nanocrystals and Co_3_O_4_–MO_x_ binary oxides for CO, CH_4_ and VOC oxidation at low temperatures: A review. Catal. Sci. Technol..

[57] Ji S.F., Xiao T.C., Wang H.T., Flahaut E., Coleman K.S., Green M.L.H. (2001). Catalytic combustion of methane over cobalt-magnesium oxide solid solution catalysts. Catal. Lett..

[58] Florea M., Matei-Rutkovska F., Postole G., Urda A., Neatu F., Pârvulescu V.I., Gelin P. (2018). Doped ceria prepared by precipitation route for steam reforming of methane. Catal. Today.

[59] Rotaru C.G., Postole G., Florea M., Matei-Rutkovska F., Pârvulescu V.I., Gelin P. (2015). Dry reforming of methane on ceria prepared by modified precipitation route. Appl. Catal. A Gen..

[60] Zeng S., Fu X., Zhou T., Wang X., Su H. (2013). Influence of pore distribution on catalytic performance over inverse CeO_2_/Co_3_O_4_ catalysts for CH_4_/CO_2_ reforming. Fuel Process. Technol..

[61] Budiman A.W., Song S.H., Chang T.S., Shin C.H., Choi M.J. (2012). Dry reforming of methane over cobalt catalysts: A literature review of catalyst development. Catal. Surv. Asia.

[62] Gil-Calvo M., Jiménez-González C., De Rivas B., Gutiérrez-Ortiz J.I., López-Fonseca R. (2017). Hydrogen production by reforming of methane over NiAl_2_O_4_/Ce_x_Zr_1-x_O_2_ catalysts. Chem. Eng. Trans..

[63] Zasada F., Grybos J., Budiyanto E., Janas J., Sojka Z. (2019). Oxygen species stabilized on the cobalt spinel nano-octahedra at various reaction conditions and their role in catalytic CO and CH_4_ oxidation, N_2_O decomposition and oxygen isotopic exchange. J. Catal..

[64] Fei Z., He S., Li L., Ji W., Au C.T. (2012). Morphology-directed synthesis of Co_3_O_4_ nanotubes based on modified Kirkendall effect and its application in CH_4_ combustion. Chem. Commun..

[65] Stefanov P., Todorova S., Naydenov A., Tzaneva B., Kolev H., Atanasova G., Stoyanova D., Karakirova Y., Aleksieva K. (2015). On the development of active and stable Pd-Co/γ-Al_2_O_3_ catalyst for complete oxidation of methane. Chem. Eng. J..

[66] Xiao T.C., Ji S.F., Wang H.T., Coleman K.S., Green M.L.H. (2001). Methane combustion over supported cobalt catalysts. J. Mol. Catal. A Chem..

[67] Chen J., Zhang X., Arandiyan H., Peng Y., Chang H., Li J. (2013). Low temperature complete combustion of methane over cobalt chromium oxides catalysts. Catal. Today.

[68] Darda S., Pachatouridou E., Lappas A., Iliopoulou E. (2019). Effect of preparation method of Co-Ce catalysts on CH_4_ combustion. Catalysts.

[69] Geng H., Yang Z., Zhang L., Ran J., Yan Y. (2017). Methane oxidation with low O_2_/CH_4_ ratios in the present of water: Combustion or reforming. Energy Convers. Manag..

