# Supplementary material for: Synthesis, Characterization and Kinetic Behavior of Supported Cobalt Catalysts for Oxidative after-Treatment of Methane Lean Mixtures
Source: Materials (Basel). 2019 Sep 27;12(19):3174. doi: 10.3390/ma12193174 (PMC6804103; doi:10.3390/ma12193174)
Supplement: Supplementary file 1 [file materials-12-03174-s001.pdf]

materials

MDPI

Supplementary Materials: Synthesis, Characterization and Kinetic Behavior of Supported Cobalt Catalysts for Oxidative After-Treatment of Methane Lean Mixtures

Andoni Choya, Beatriz de Rivas, Jose Ignacio Gutiérrez-Ortiz, Juan Ramón González-Velasco and Rubén López-Fonseca *

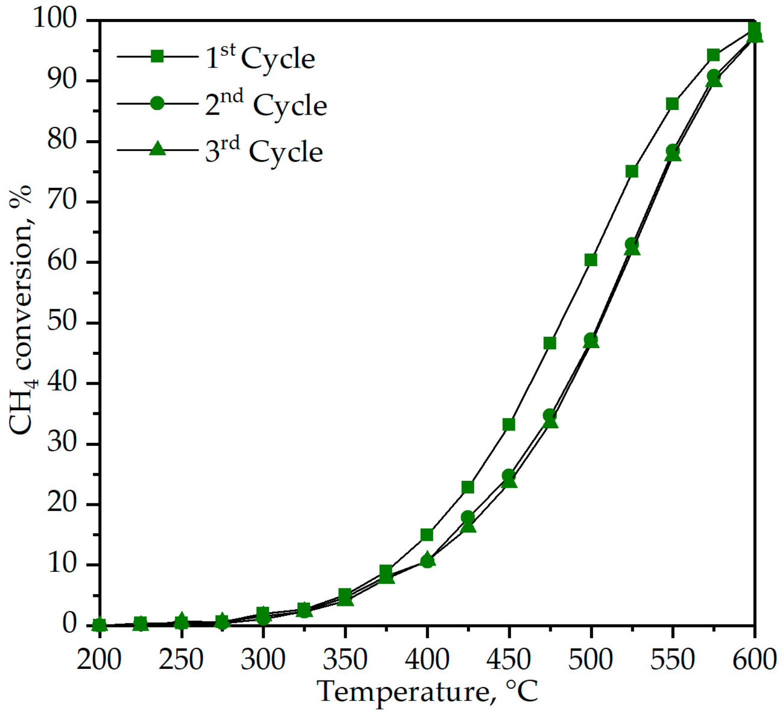

Figure S1. Light-off cycles of the Co/CeO_2 catalyst.

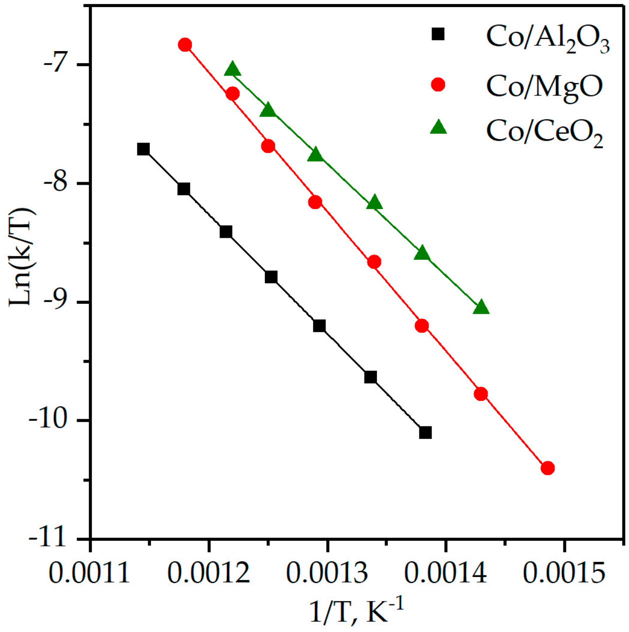

Figure S2. Linearized plots of the Eyring-Polanyi equation.
